# Acute Occlusion of the Infarct-Related Artery as a Predictor of Very Long-Term Mortality in Patients with Acute Myocardial Infarction

**DOI:** 10.1155/2021/6647626

**Published:** 2021-11-24

**Authors:** Nikola Kos, Ivan Zeljković, Tomislav Krčmar, Karlo Golubić, Fran Šaler, Marijan Erceg, Diana Delić-Brkljačić, Nikola Bulj

**Affiliations:** ^1^Department of Cardiovascular Diseases, University Hospital Centre Sestre Milosrdnice, Zagreb, Croatia; ^2^Department of Cardiovascular Diseases, University Hospital Centre Zagreb, Zagreb, Croatia; ^3^Department of Cardiovascular Diseases, University Hospital Centre Rijeka, Rijeka, Croatia; ^4^Department of Cardiovascular Diseases, University Hospital Dubrava, Zagreb, Croatia; ^5^Croatian Institute of Public Health, Zagreb, Croatia; ^6^School of Medicine, University of Zagreb, Zagreb, Croatia

## Abstract

**Aim:**

The survey's aim was to examine the significance of infarct-related artery (IRA) occlusion (verified angiographically) on very long-term outcomes of patients with acute myocardial infarction, within the STEMI and NSTEMI diagnosis.

**Methods:**

A single-center, nonrandomized, registry-based study on patients treated for acute coronary syndrome with percutaneous coronary intervention between June 2011 and December 2016 was conducted. Patients with angiographically proven IRA occlusion (100% stenosis with TIMI flow 0 distal to occlusion) were categorized as occlusive myocardial infarction (OMI) and patients with patent IRA (50–99% stenosis with TIMI 1–3 flow) were categorized as nonocclusive myocardial infarction (NOMI) and very long-term outcomes were analyzed. Data were collected prospectively from the hospital's PCI registry and the database of the Croatian Institute of Public Health.

**Results:**

A total of 2450 patients were included in the study. 796 (32.5%) patients had NOMI and 1654 patients (67.5%) had OMI. According to ECG changes, 1534 patients presented with STEMI (62,6%) and 916 with NSTEMI (37,8%). 88% of STEMI patients presented with OMI and 12% with NOMI, while patients with NSTEMI in 33,8% presented with OMI and in 66,81% with NOMI. A median follow-up was 4.7 years. There was no significant difference in cardiovascular mortality between the groups (14.8% vs 13.1%; OMI vs NOMI, respectively; *p*=0.374) neither in all-cause mortality (19% vs 21.5%; OMI vs NOMI, respectively; *p*=0.374). Patients with NSTEMI had a significantly higher very long-term mortality (21.6% vs 18.1%; NSTEMI vs STEMI, respectively; *p*=0.029).

**Conclusion:**

The main findings of the study are as follows: (1) total IRA occlusion was not associated with higher long-term mortality; (2) NSTEMI was associated with a higher mortality rate compared with STEMI, independent of angiographic presentation (OMI/NOMI); (3) IRA occlusion was not associated with significantly higher mortality rates in patients with STEMI and NSTEMI, respectively.

## 1. Introduction

The pathophysiology of acute myocardial infarction with ST elevation (STEMI) and without ST elevation (NSTEMI) differs significantly. Namely, STEMI is usually caused by a complete occlusion of the infarct-related artery (IRA), which is not the case with NSTEMI which is mostly caused by transient or incomplete IRA occlusion [1, 2]. Patients with STEMI have a worse short-term (30 days) prognosis, but if they overcome the acute phase, they tend to have a better long-term survival in comparison to NSTEMI patients [3, 4]. Although there are many theories explaining why NSTEMI patients have a higher long-term mortality rate, there are few dedicated studies evaluating the impact of the pathophysiological basis of infarction on outcome [5–7]. Additionally, electrocardiogram (ECG) is insufficient in detecting artery occlusion and therefore dividing patients according to IRA patency would give more precise information about patient outcomes [8, 9]. This study aims to examine the significance of IRA occlusion (verified angiographically) on the very long-term outcomes of patients with acute myocardial infarction, within the STEMI and NSTEMI diagnosis.

## 2. Methods

We conducted a single-center, nonrandomized, registry-based study. Patients treated with primary percutaneous coronary intervention (PCI) due to acute myocardial infarction, between June 2011 and December 2016, were included in the study. Demographic, laboratory, echocardiographic, and angiographic data as well as data on long-term mortality were collected from the hospital's PCI registry and the database of the Croatian Institute of Public Health. Patients with acute myocardial infarction treated medically or not undergoing primary PCI, as well as patients with incomplete medical documentation, were excluded from the study. All coronary angiography procedures were performed according to valid international guidelines and performed by nine high-volume interventional cardiologists. [10] The time period within the PCI was performed following current ESC guidelines' recommendation (120 minutes from the first medical contact for STEMI patients and according to the risk stratification for NSTEMI patients). [11] Patients with angiographically proven IRA occlusion (100% stenosis with TIMI flow 0 distal to occlusion) were categorized as occlusive myocardial infarction (OMI) and patients with patent IRA (50–99% stenosis with TIMI 1–3 flow) were categorized as nonocclusive myocardial infarction (NOMI). Patients were pretreated with medications according to valid ESC guidelines at the time of the intervention (unfractionated heparin 80 IJ/kg; acetylsalicylic acid (ASA) 300 mg as loading dose followed by 100 mg daily lifelong + clopidogrel 600 mg or ticagrelor 180 mg as loading dose followed by clopidogrel 75 mg or ticagrelor 2 × 90 mg daily up to one year). [11] The follow-up period lasted until January 1, 2019.

The primary outcome was defined as cardiovascular mortality during the follow-up period and the secondary outcome as all-cause mortality during the follow-up period.

### 2.1. Statistical Analysis

Categorical variables are presented as absolute values and percentages. Categorical variables were compared by the chi-square test. Continuous data are expressed as means and standard deviations or median with corresponding interquartile range (IQR). For continuous variables, comparisons were made using student's *T*-test or Mann–Whitney *U* test, as appropriate. Cox regression (proportional hazards) regression was used to examine the independence of prognostic factors. The Kaplan–Meier test was used to calculate the survival rate, and the log-rank test was used to compare the survival rate between groups. *p* values <0.05 were considered significant. The statistical analysis was performed using SPSS Version 20 (IBM SPSS Statistics, New York, USA).

## 3. Results

A total of 2450 patients were included in the study. 796 (32.5%) patients had NOMI and 1654 patients (67.5%) had OMI. Baseline characteristics and laboratory and angiographic findings of the two studied groups are shown in Table 1. No significant interoperator mortality difference was observed. Almost all patients were pretreated with 300 mg of acetylsalicylic acid (99,9%) and most of OMI (1487/1654; 90,5%) and NOMI (772/796; 96,7%) patients were pretreated with 600 mg of clopidogrel; the rest were pretreated with ticagrelor 180 mg. All patients were recommended to take the dual antiplatelet therapy (ASA 100 mg + clopidogrel 75 mg/ticagrelor 2 × 90 mg) for one year (according to our national healthcare insurance guidelines, it was not possible to prescribe a dual therapy for more than a year), continued by ASA 100 mg lifelong.

The OMI patients were significantly younger (63 (55–73) vs 66 (58–75) for NOMI (*p* < 0.001)), more often male (72,9% vs 27,1%; *p* < 0.000) and smokers (40,9% vs 34,9%; *p* < 0.001), while NOMI patients more often had diabetes (29.6% vs 20.9%; *p* < 0.001) and multivascular diseases (peripheral arterial disease: 8.3% vs 5.4% (*p*=0.005); carotid stenosis: 6.8% vs 5.4% (*p* < 0.001)).

According to ECG changes, 1534 patients presented with STEMI (62,6%) and 916 with NSTEMI (37,8%). 88% of STEMI patients presented with OMI and 12% with NOMI, while patients with NSTEMI in 33,8% presented with OMI and in 66,81% with NOMI.

SYNTAX score was significantly higher in patients with NOMI (23 (18–27) vs. 21 (17–26); *p* < 0.001), and in the same group, significant stenosis of the LMCA was found more frequently (10,1% vs. 3,6%; *p* < 0.001).

**Figure 1:**
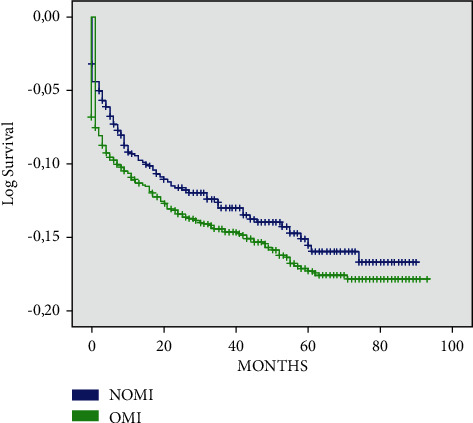
Cardiovascular mortality during follow-up period. NOMI, nonocclusive myocardial infarction; OMI, occlusive myocardial infarction.

After a median follow-up of 4.7 years, 348 patients died from cardiovascular etiology (14.2%). There was no significant difference between the two studied groups (14.8% vs 13.1%; OMI vs NOMI, respectively; *p*=0.374). Throughout follow-up, higher mortality was observed in patients with OMI with early curve separation within one month (Figure 1).

**Figure 2:**
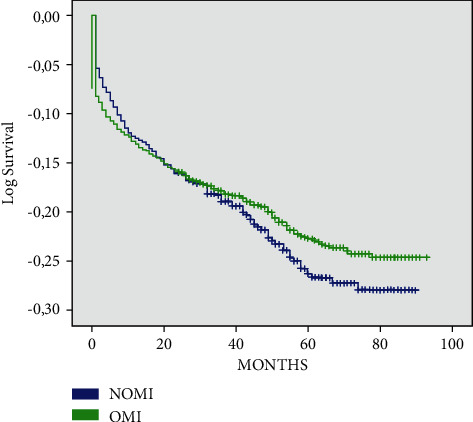
All-cause mortality during follow-up period. NOMI, nonocclusive myocardial infarction; OMI, occlusive myocardial infarction.

The total all-cause mortality rate was 477 (19.5%). The number of deaths during follow-up did not differ significantly between the groups with OMI (19%) and NOMI (21.5%) (*p*=0.374) (Figure 2).

In the observed period, patients with NSTEMI had a significantly higher very long-term mortality (21.6% vs 18,1%; NSTEMI vs STEMI, respectively; *p*=0.029). In patients with STEMI and OMI, the mortality rate was 18.1% and in the group with STEMI and NOMI was 17.9% (*p*=0.687). Nonsignificantly higher mortality was present in patients with NSTEMI and NOMI (22.3%) vs NSTEMI and OMI (21.1%) (*p*=0.927).

The use of thrombus aspiration or adding GP IIb/IIIa inhibitor on top of standard therapy did not have a significant impact on outcomes (*p*=0,341 and *p*=0,14 for use of thrombus aspiration and GP IIb/IIIa inhibitors, respectively).

Multivariant analysis indicated that age, peripheral artery disease, admission level of glucose, ventricular tachycardia during PCI, ejection fraction of the left ventricle, severe mitral regurgitation, and aortic stenosis were independent predictors for patient long-term mortality (Table 2). Arterial hypertension or atrial fibrillation did not independently affect the primary outcome.

## 4. Discussion

The main findings of this registry-based study, involving 2450 patients and with the median follow-up being 4.7 years, are as follows: (1) total IRA occlusion was not associated with higher long-term cardiovascular mortality (14.8% vs 13.1%; OMI vs NOMI, respectively; *p*=0.374); (2) NSTEMI was associated with higher mortality rate compared with STEMI, independent of angiographic presentation (OMI/NOMI) (21.6% vs 18,1%; NSTEMI vs STEMI, respectively; *p*=0.029); (3) IRA occlusion was not associated with a significantly higher mortality rates in patients with STEMI and NSTEMI, respectively.

Up to our very best knowledge, this is the first study which compared very long-term outcomes in patients with acute myocardial infarction exclusively based on IRA patency. We found no difference between the OMI and NOMI groups in terms of very long-term mortality. This finding is not in correlation with a traditional ECG-based diagnosis (where IRA occlusion is more often associated with STEMI and patent IRA with NSTEMI) where NSTEMI patients tend to have higher long-term mortality. The mentioned is proved in our study [12].

Our results show that 12% of patients with STEMI have a patent IRA and 33,8% of NSTEMI patients have occluded IRA, which is consistent with previous published studies [6, 7, 13, 14]. It is known that part of STEMI patients with occluded IRA develop a “spontaneous reperfusion” of the IRA (probably due to antiplatelet therapy or after the first “flash” of contrast during coronary angiography). [15] Probably some patients with nonocclusive STEMI are actually spontaneously recanalized occlusive STEMI. The outcome for STEMI patients does not depend on whether they presented as OMI or NOMI, which partially confirms the theory of spontaneous recanalization.

The accuracy of the 12-channel electrocardiogram in detecting coronary artery occlusion is limited [16]. Approximately every fifth patient with NSTEMI has a totally occluded IRA [8]. The available data from several registry-based and randomized studies on the impact of occluded IRA on NSTEMI patient's outcomes are contradictory, majority found no association between IRA occlusion and worse outcome in NSTEMI patients [5, 6, 14, 17–19]. Our study showed that an occluded IRA was found in 33,18% of NSTEMI patients, but with no effect on long-term outcomes compared to NSTEMI patients with patent IRA. Compared to other studies, our study had the longest follow-up period and among the highest number of included patients.

Patients with NOMI are significantly burdened with comorbidities (multivessel disease, diabetes, peripheral arterial disease, arterial hypertension, and previous myocardial infarction), which affects their subsequent mortality and finally equates long-term mortality of OMI patients.

Results of the present study should be interpreted in the light of several limitations. Firstly, the study design was unicentric and observational, and patients with acute myocardial infarction treated with modalities other than PCI (eg., cardiac surgery) were not included; patients treated medically and with fibrinolysis were excluded from the study. This study included only patients with performed angiography and did not include patients who died in outpatient settings or did not undergo coronary angiography, so these results should not be extrapolated into the mentioned groups. Secondly, there is a lack of information about patients' compliance to recommended therapy, shortening of prescribed one-year DAPT therapy, and algorithms of “triple” therapy (DAPT + OAC). Thirdly, it was not possible to compare troponin values due to multiple cardiac troponin assay changes.

## Figures and Tables

**Table 1:** Demographic, laboratory, echocardiographic, and angiographic data and procedural characteristics.

	OMI	NOMI	*p* value
*Basic characteristics and comorbidities*			
Age (years, median (IQR))	63 (55–73)	66.3 (58–75)	<0.0001
Male (no., %)	1206 (72,9)	572 (27,1)	0.584
BMI (kg/m^2^, median (IQR))	27,8 (25,3–30,9)	27,8 (25–30,5)	0.388
STEMI (no., %)	1350 (81,6)	184 (23,1)	<0.0001
Grace (no., median (IQR)	141,5 (96–175)	144 (106–75)	0.3
Atrial fibrillation (no., %)	95 (5,7)	65 (8,2)	0.018
Arterial hypertension (no., %)	1176 (71,1)	624 (78,4)	<0.001
Hyperlipoproteinemia (no., %)	772 (46,7)	384 (48,2)	0.89
Diabetes mellitus (no., %)	346 (20,9)	236 (29,6)	<0.001
Family history of IHD (no., %)	545 (33)	280 (35,2)	0.649
Smoking history (no., %)	676 (40,9)	278 (34,9)	<0.001
Peripheral artery disease (no., %)	89 (5,4)	66 (8,3)	0.005
Carotid stenosis (no., %)	60 (3,6)	54 (6,8)	<0.001
Previous myocardial infarction (no., %)	166 (10)	177 (14,7)	<0,001
Previous coronary intervention (no., %)	140 (8,5)	89 (11,2)	0,03
Previous stroke (no., %)	83 (5)	46 (5,8)	0,427
Previous coronary bypass surgery (no., %)	26 (1,6)	14 (1,8)	0,735

*Hospital admission data*			
Cardiogenic shock at admission (no., %)	107 (6,5)	19 (2,4)	<0,001
Ventricular tachycardia before or during PCI (no., %)	48 (2,9)	17 (2,1)	0,028
Ventricular fibrillation before or during PCI (no., %)	129 (7,8)	16 (2)	<0,001
Out-of-hospital arrest survivors (no., %)	62 (3,7)	12 (1,5)	0,003

*Angiographic data*			
SYNTAX (no., median (IQR))	21 (17–26)	23 (18–27)	<0.001
PCI LMCA (no., %)	25 (1,5)	33 (4,1)	<0.001
PCI proximal LAD (no., %)	371 (22,4)	168 (21,1)	0.305
LMCA stenosis (no., %)	60 (3,6)	80 (10,1)	<0.001
Proximal LAD stenosis (no., %)	453 (27,4)	233 (29,3)	0.43

*Laboratory and echocardiographic findings*			
Hemoglobin (x10^12^/L, median (IQR))	143 (131–153)	141 (129–150)	0.003
Leukocytes (x10^9^/L, median (IQR))	10,6 (8,5–13,2)	9 (7,4-11-2)	<0.001
Platelets (x10^9^/L, median (IQR))	218 (183–261)	215 (179–257)	0.054
Creatinine (*μ*mol/L, median (IQR))	90 (75–108)	90 (75–105)	0.846
eGFR (mL/min/1,73 m^2^, median (IQR))	73 (59–88)	73 (58–87)	0.539
Glucose (mmol/L, median (IQR))	7,6 (6,2–10,2)	6,8 (5,7–9,1)	<0.001
Total cholesterol (mmol/L, median (IQR))	5,2 (4,3–6,2)	4,9 (4–5,8)	<0.001
HDL (mmol/L, median (IQR))	1,1 (0,9–1,2)	1 (0,8–1,2)	0.001
LDL (mmol/L, median (IQR))	3,5 (2,7–4,2)	3,1 (2,3–3,9)	<0.001
Triglycerides (mmol/L, median (IQR))	1,5 (1–2,1)	1,4 (1,1–2,1)	0.011
CK, admission value (U/L, median (IQR))	209 (114–539)	161 (85–366)	<0.001
CPK, maximal value (U/L, median (IQR))	1629 (716–3406)	354 (163–812)	<0.001
LDH (U/L, median (IQR))	229 (183–331)	221 (181–292)	0.012
CRP (mg/L, median (IQR))	4,7 (1,9–14,5)	5,8 (2,2–16,7)	0.024
Left ventricular ejection fraction (%, median (IQR))	50 (45–57)	55 (45–60)	<0,001
Severe aortic stenosis (no., %)	6 (0,4)	15 (1,9)	0.002
Severe mitral regurgitation (no., %)	52 (3,1)	45 (5,7)	0.004

**Table 2:** Multivariant analysis.

Variable	HR	95% CI	*p* value
Age	1,067	1,052–1,084	0
STEMI (no., %)	1,067	0,431–2,922	0,542
Atrial fibrillation (no., %)	1,832	0,527–1,537	0,176
Arterial hypertension (no., %)	1,832	0,623–1,421	0,68
Diabetes mellitus (no., %)	3,266	0,923–6,431	0,071
Smoking history (no., %)	0,433	0,230–1,021	0,805
Peripheral artery disease (no., %)	0,523	0,356–0,770	0,001
Carotid stenosis (no., %)	1,949	0,924–1,794	0,163
Previous myocardial infarction (no., %)	1,933	0,412–2,530	0,164
Previous coronary intervention (no., %)	1,997	0,876–2,233	0,158
Cardiogenic shock at admission (no., %)	1,738	0,764–2,325	0,187
Ventricular tachycardia before or during PCI (no., %)	0,253	0,133–0,481	0
Ventricular fibrillation before or during PCI (no., %)	0,003	0,012–2,124	0,96
Out-of-hospital arrest survivors (no., %)	0,282	0,123–4,344	0,596
SYNTAX (no., median (IQR))	0,305	0,120–2,311	0,581
PCI LMCA (no., %)	3,036	0,937–4,305	0,081
LMCA stenosis (no., %)	0,109	0,023–1,753	0,741
Hemoglobin (x10^12^/L, median (IQR))	0,988	0,981–0,995	0,001
Leukocytes (x10^9^/L, median (IQR))	0,698	0,252–3,591	0,967
Glucose (mmol/L, median (IQR))	1,051	1,030–1,073	0
Total cholesterol (mmol/L, median (IQR))	0,933	0,974–1,522	0,073
HDL (mmol/L, median (IQR));	0,152	0,098–1,101	0,696
LDL (mmol/L, median (IQR))	3,098	0,699–5,343	0,078
Triglycerides (mmol/L, median (IQR))	1,401	0,984–1,732	0,237
CK, admission value (U/L, median (IQR))	0,578	0,345–1,545	0,447
CK, maximal value (U/L, median (IQR))	2,982	0,923–5,830	0,084
LDH (U/L, median (IQR))	0,538	0,212–2,345	0,463
CRP (mg/L, median (IQR))	1,159	0,714–1,953	0,282
Left ventricular ejection fraction (%, median (IQR))	0,967	0,953–0,982	0
Severe aortic stenosis	0,476	0,229–0,989	0,027
Severe mitral regurgitation	0,6	0,405–0,899	0,011

Continuous variables are presented as median values with associated interquartile ranges. Categorical variables are expressed in absolute and relative frequencies. Cox regression (proportional hazards) regression was used to examine the independence of prognostic factors. HR, hazard ratio; 95% CI, confidence interval; *p* values <0.05 were considered significant; IQR, interquartile range; STEMI, ST elevation myocardial infarction; PCI, percutaneous coronary intervention; SYNTAX, SYNergy between percutaneous coronary intervention with TAXus and cardiac surgery; LMCA = left main coronary artery; LAD = left anterior descending artery; HDL = high-density lipoproteins; LDL = low-density lipoproteins; CK = creatine kinase; LDH = lactate dehydrogenase; CRP = C-reactive protein.

## Data Availability

The core data used to support the findings of this study have been deposited in the hospital's internal database and registry and the database of the Croatian Institute of Public Health. Moreover, summarized data used to support the findings of this study are included in this article. The individual data used to support the findings of this study are restricted by the Croatian law to protect patients' privacy. Data are available from Nikola Kos, MD, corresponding author, for researchers who met the criteria for access to confidential data.
